# The Role of Connexins in Wound Healing and Repair: Novel Therapeutic Approaches

**DOI:** 10.3389/fphys.2016.00596

**Published:** 2016-12-06

**Authors:** Pui Wong, Teresa Tan, Catherine Chan, Victoria Laxton, Yin Wah Fiona Chan, Tong Liu, Wing Tak Wong, Gary Tse

**Affiliations:** ^1^Li Ka Shing Faculty of Medicine, School of Biomedical Sciences, University of Hong KongHong Kong, Hong Kong; ^2^Department of Surgery, Faculty of Medicine, Chinese University of Hong KongHong Kong, Hong Kong; ^3^Intensive Care Department, Royal Brompton and Harefield NHS Foundation TrustLondon, UK; ^4^Department of Psychology, School of Biological Sciences, University of CambridgeCambridge, UK; ^5^Tianjin Key Laboratory of Ionic-Molecular Function of Cardiovascular Disease, Department of Cardiology, Tianjin Institute of Cardiology, Second Hospital of Tianjin Medical UniversityTianjin, China; ^6^School of Life Sciences, Chinese University of Hong KongHong Kong, Hong Kong; ^7^Department of Medicine and Therapeutics, Faculty of Medicine, Chinese University of Hong KongHong Kong, Hong Kong; ^8^Faculty of Medicine, Li Ka Shing Institute of Health Sciences, Chinese University of Hong KongHong Kong, Hong Kong

**Keywords:** gap junctions, connexins, antisense oligodeoxynucleotides, connexin mimetic peptides, gap junction modulators, wound healing, wound repair

## Abstract

Gap junctions are intercellular proteins responsible for mediating both electrical and biochemical coupling through the exchange of ions, second messengers and small metabolites. They consist of two connexons, with (one) connexon supplied by each cell. A connexon is a hexamer of connexins and currently more than 20 connexin isoforms have been described in the literature thus far. Connexins have a short half-life, and therefore gap junction remodeling constantly occurs with a high turnover rate. Post-translational modification, such as phosphorylation, can modify their channel activities. In this article, the roles of connexins in wound healing and repair are reviewed. Novel strategies for modulating the function or expression of connexins, such as the use of antisense technology, synthetic mimetic peptides and bioactive materials for the treatment of skin wounds, diabetic and pressure ulcers as well as cornea wounds, are considered.

## Introduction

Gap junctions are intercellular channels that mediate both electrical and biochemical coupling through the exchange of ions, second messengers, and small metabolites (Kanno and Loewenstein, 1964; Lawrence et al., 1978). Gap junction intercellular communication (GJIC) is essential for the regulation of cellular differentiation and apoptosis, movement of cells within tissues, and intracellular signaling (Zhou and Jiang, 2014). In excitable tissues, GJIC also governs the conduction of electrical signals between successive cells (Koval et al., 2014; Veeraraghavan et al., 2014, 2015; Tse, 2016; Tse et al., 2016a). A gap junction is formed by two connexons, where one is provided by each cell (Harris, 2001). Each connexon is a hexamer of connexins (Cx). Currently 21 members of the human connexin gene family have been identified (Söhl and Willecke, 2004).

Some connexin isoforms are cell-type specific, and their expression is induced by different metabolic states, such as pluripotent stem cell induction (Ke et al., 2013), epidermal wound healing (Becker et al., 2012), epithelial-to-mesenchymal transition (EMT) (Zhou and Jiang, 2014), and pathological states such as hepatitis (Crespo Yanguas et al., 2016). Connexins can be found in both excitable and non-excitable tissues with different spatio-temporal patterns. For example, the cardiac myocardium has abundant expression of the isoforms Cx30.2, Cx40, Cx43, and Cx45 (Davis et al., 1995; Jongsma, 2000; Tse and Yeo, 2015). Their expression levels vary between different cardiac regions: Cx40 is only expressed in the atria; whereas in the ventricles Cx43 is extensively expressed with minimal levels of Cx40. During cardiac development, Cx45 levels are progressively downregulated (Alcoléa et al., 1999). In non-excitable tissue, Cx43 can be found in breasts, kidneys, skin and lungs; Cx26 is expressed in liver, kidneys and oesophageal epithelium, and Cx32 is found in liver and kidneys (Wilgenbus et al., 1992; Goldberg et al., 2004).

Gap junctions operate through two distinct gating mechanisms: membrane voltage-dependent and transjunctional voltage-dependent gating (also known as fast and slow gating; Bukauskas and Verselis, 2004). Besides voltage sensitivity, both mechanosensitivity and chemosensitivity have been reported (Bao et al., 2004; Bukauskas and Verselis, 2004). Connexin activity is influenced by intracellular Ca^2+^, pH, chemical uncouplers (Tse et al., 2016b,c,d,e,f), phosphorylation events (Musil and Goodenough, 1991; Bennett and Verselis, 1992), and lipid availability in the immediate environment, including low-density lipoprotein, apolipoprotein-B (Meyer et al., 1991) and cholesterol (Meyer et al., 1990). Gap junctions allow the passive diffusion of ions, intracellular molecules that include metabolites and messengers such as cyclic AMP, cyclic GMP and IP_3_. Undocked connexons are not inactive, but can participate in intracellular signaling (Evans et al., 2006). Transient opening of connexons can permit entry of extracellularly released molecules during cellular stress (Froger et al., 2010), whereas prolonged opening may initiate cell death pathways.

In recent years, there has been growing interest in the role of connexins and therapeutic usage of gap junction modulators in various clinical conditions (O'Carroll et al., 2013). As well as modifying gap junction function, other different interventions can alter the synthesis, transport, assembly, phosphorylation, and degradation of gap junction proteins (Beyer and Berthoud, 2002). Gene therapy can restore or increase GJIC in transfected cells and “knock-in” animals (Plum et al., 2000; Beyer and Berthoud, 2002). The different treatment options in the experimental stages are presented in Table 1. This article will focus on the roles of gap junctions in wound healing while also discussing potential directions for further investigation and treatment development.

**Table 1:** **Vehicles used include Pluronic Gel and microcapsules**.

**Class**	**Mechanism**	**Examples**	**Disease**	**References**
Antisense oligodeoxynucleotides	Binding to messenger RNA encoding for connexins	Cx43-specific antisense oligodeoxynucleotides (Cx43 AsODN)	Skin wound healing	Qiu et al., 2003; Mori et al., 2006; O'Carroll et al., 2013
Connexin mimetic peptides	Direct binding to connexins	αCT1	Skin wound healing; Diabetic foot ulcers; venous leg ulcers; corneal wound healing	Moore et al., 2013; Grek et al., 2014, 2015; Ghatnekar et al., 2015
		Gap27	Skin wound healing; pressure ulcers; Diabetic foot ulcers	Evans and Boitano, 2001; Pollok et al., 2011
Biomaterials	Alteration of gap junction behavior, and upregulation of growth factors	Bioactive glass	Skin wound healing	Li et al., 2016

## Skin wound healing

**Figure 1:**
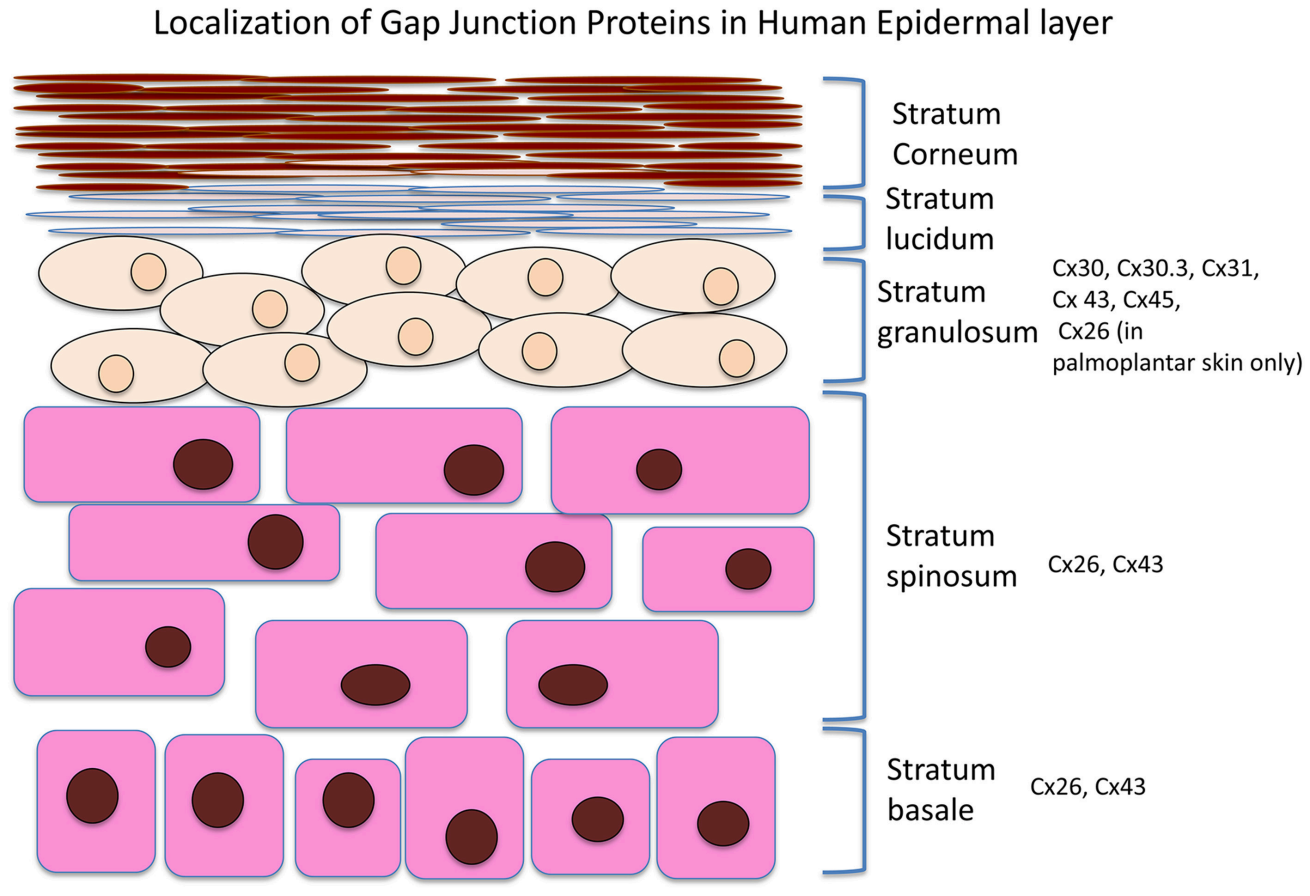
**Distribution of different connexin isoforms in different layers of the skin**.

The integumentary system is the largest system of the body and maintenance of its integrity is critical to survival of the organism. A number of connexins can be found in the skin, including Cx26, 30, 30.3, 31, 31.1, 32, 37, 40, 43, and 45. An overview of the Cx expression patterns in the different skin layers is presented in Figure 1. Cx43, the predominant isoform found in skin, is mainly expressed in the strata spinosum and basale, whereas Cx26 is detected in the basal layers and upper stratum spinosum (Wiszniewski et al., 2000; Wang et al., 2010). Of these, Cx43 localizes to the skin vasulature, fibroblasts, dermal appendages and the basal and lower spinous layers (Mendoza-Naranjo et al., 2012), It can interact with different components in tight and adherens junctions (Scott et al., 2012). Tight junctions are made of proteins such as zona occludens-1 and -2 (ZO-1, ZO-2), and have a barrier function to prevent passage of molecules and ions between plasma membranes of adjacent cells (Kirschner and Brandner, 2012). Cx43 interacts with ZO-1 and -2 in a cell cycle phase-specific manner, thereby regulating cell growth, differentiation, migration, and proliferation (Singh et al., 2005).

Skin wound healing, which occurs in response to injury, involves a complex interplay of physiological processes (Bajpai et al., 2009). Optimal wound healing can be divided into the following four stages of hemostasis, inflammation, proliferation and maturation. Angiogenesis, re-epithelialization and collagen repair are essential, taking place mainly during the latter part of wound healing, proliferation and maturation stages (Guo and Dipietro, 2010). Connexins are present in both the dermis and epidermis (Ghatnekar et al., 2009), with the exception of the uppermost layer of the epidermis, the stratum corneum (Caputo and Peluchetti, 1977; Scott et al., 2012). The distribution of connexin isoforms varies throughout the epidermis, and potentially plays a role in regulating keratinocyte differentiation (Brissette et al., 1994; Lucke et al., 1999). Inflammatory and growth factors can pass through gap junctions to exert their effects on target sites.

**Figure 2:**
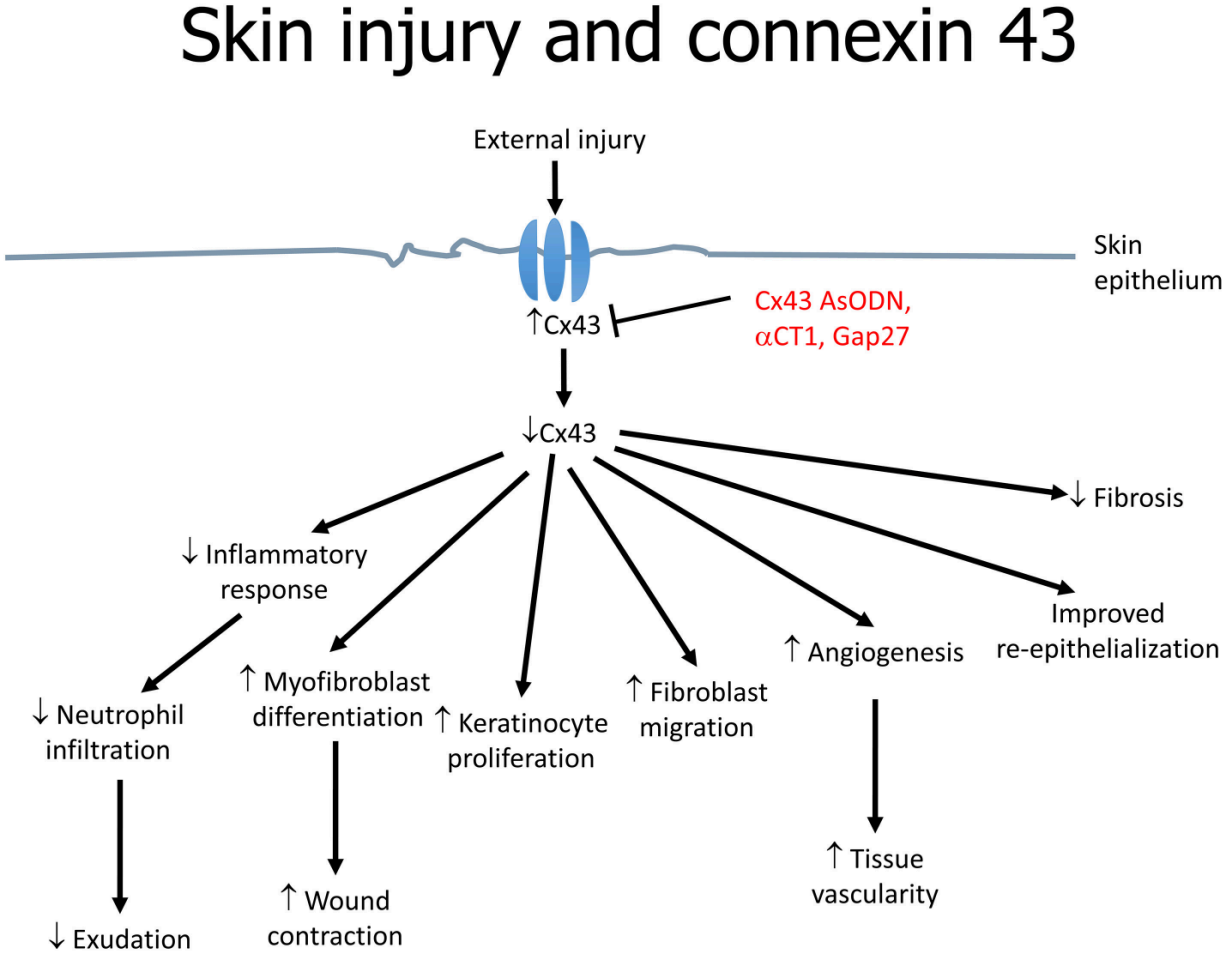
**The role of connexin 43 in skin wound healing in response to injury**.

Cx43 downregulation is associated with increased angiogenesis, migration of fibroblasts and multiplication of keratinocytes as well as reduced infiltration of immune cells (Grek et al., 2014) (Figure 2). These effects are mediated by the upregulation of transforming growth factor-beta (TGF-β) and collagen α1, and the downregulation of the inflammatory mediators, chemokine (C-C motif) ligand 2 (CCL2) and tumor necrosis factor-alpha (TNF-α) (Grek et al., 2015). TGF-β3 is of interest as it has been shown to accelerate wound healing time and scarring, which was associated with decreased Cx43 (Jin et al., 2008). TGF-β1 is closely associated with skin wound healing; its expression is upregulated in a fibroblast wound-healing model involving Cx43 knockdown (Mori et al., 2006). For example, in an autosomal dominant disorder termed oculodentodigital dysplasia (ODDD), skin manifestations are associated with over 70 mutations in the Cx43 gene (GJA1) (Esseltine et al., 2015). In a mouse model with a G60S mutation in GJA1, a delay in wound closure compared to wild-type littermates is observed, which is likely to be due to defects in the dermal fibroblasts. Indeed, in dermal fibroblasts obtained from patients with ODDD, GJIC is reduced (Churko et al., 2011).

Cx43 proteins can exist in multiple phosphorylated forms (Budunova et al., 1994; Kuroki et al., 1998). Serine phosphorylation of the C-terminal can alter channel gating, thereby regulating GJIC (Márquez-Rosado et al., 2012). For example, S373 phosphorylation disrupts Cx43 interaction with ZO-1, which promoted Cx43 accumulation and assembly into larger gap junctions, in turn enhancing GJIC (Solan and Lampe, 2014). TPA enhances Cx43 phosphorylation on the S368 residue via PKC (Márquez-Rosado et al., 2012). Consequently, reduced channel conductance and GJIC were observed (Lampe et al., 2000; Solan et al., 2003). Other post-translational modification events can be exemplified by S279/282 phosphorylation leading to gap junction closure (Lin et al., 2001). S368 phosphorylation of Cx43 in the basal cell compartment, which peaks at 24 h (Márquez-Rosado et al., 2012), led to reduced GJIC and enhanced migration of keratinocytes (Richards et al., 2004). The phosphorylation levels return to baseline at 72 h after initial skin injury. Cx43 phosphorylation and TGF-β1 also contribute to the transformation of fibroblasts into myofibroblasts, which are responsible for wound contraction and thus improved healing results (Churko and Laird, 2013). These findings demonstrate therapeutic potential in regulation of Cx43 through modulating upstream pathways responsible for Cx43 phosphorylation at different amino acid residues. However, to fulfill its full therapeutic potential for adequate design of kinase-targeting drugs, a deeper knowledge of the kinase system is required (Solan and Lampe, 2014).

**Figure 3:**
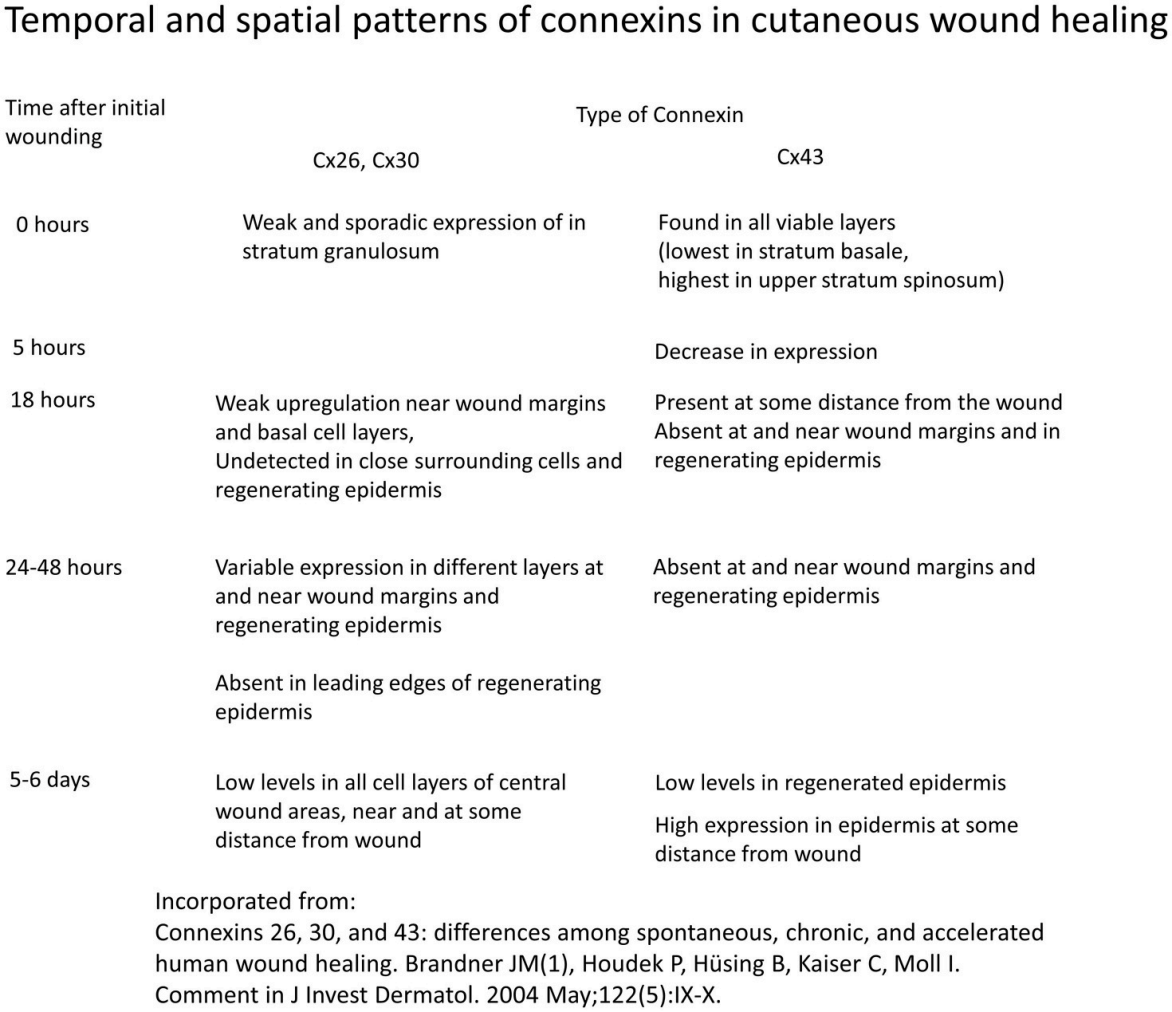
**Temporal and spatial patterns of connexins in cutaneous wound healing**. Information derived from Brandner et al. (2004).

Different spatio-temporal patterns of connexins expression have been observed in various stages of wound healing (Figure 3; Coutinho et al., 2003; Brandner et al., 2004). Initially, all connexins are downregulated in response to wounding (Coutinho et al., 2003). In mouse models, Cx26 and Cx30 are upregulated in epidermal cells proximal to the wound, but are downregulated in cells around the edge (Coutinho et al., 2003; Becker et al., 2012). A similar pattern was found in human cutaneous wound healing, with initial absence of staining of Cx26, Cx30, and Cx43 around the wound site (Brandner et al., 2004). This altered distribution pattern is most pronounced at 24 h. At the later resolution stage, Cx26 remains abundantly expressed, whereas Cx31.1 and Cx43 returns to normal pre-wounded levels (Goliger and Paul, 1995; Brandner et al., 2004; Becker et al., 2012).

A number of approaches can be used to alter gap junctions. The first is antisense technology. Cx43-specific antisense oligodeoxynucleotides (Cx43 AsODN) incorporated into Pluronic Gel have been tested on cutaneous wounds. The Pluronic Gel consists of both aqueous and organic phases within a micellar network and can efficiently partition with the skin, facilitating transport of pharmacological active substances across the skin. This approach led to suppression of Cx43 expression and improvements in both the rate and quality of healing (Qiu et al., 2003; Mori et al., 2006). The most commonly used AsODN is an ssDNA that consists of 30 deoxynucleotides with an unmodified backbone (O'Carroll et al., 2013). This allows direct inhibition of Cx43 translation by complementary binding to the messenger RNA, hence depleting the cells of Cx43. As a result, the level drops since it is being continuously degraded by proteasomes. Multiple events findings were noted as a consequence: (1) inflammatory response attenuation; (2) enhanced myofibroblast differentiation and wound contraction; (3) increased keratinocyte proliferation; (4) fibroblast migration; (5) increased rate of angiogenesis (6) improved re-epithelialization and granulation tissue formation (Figure 2). On a macroscopic level, the wounds demonstrated a reduction in inflammation and exudation (Qiu et al., 2003). Neutrophil infiltration is part of the immune response against potential pathogen invasion of the wound site, but may also delay wound closure (Dovi et al., 2003). Cx43 AsODN treatment led to reductions in the number of neutrophils and levels of cytokines such as TNF-α and CCL2 at the injury site, and may accelerate epidermal healing (Rossi and Zlotnik, 2000). Loss of Cx43 increases the speed of wound closure (Kretz et al., 2003; Qiu et al., 2003). This may favor keratinocyte mobilization, proliferation and transformation into a migratory phenotype. However, re-expression of Cx43 is important in post-wound stages. Therefore, the importance of time-dependent regulation of gap junction expression during wound healing should be recognized.

Another approach to alter gap junction function is the application of mimetic peptides. These are synthetic compounds with sequence homologies to a short conserved extracellular loop domain of connexins (Desplantez et al., 2012), and can reversibly inhibit GJIC (O'Carroll et al., 2013). An example is the alpha-carboxy terminus 1 (αCT1), which can inhibit Cx43 by competitive binding to ZO-1 (Grek et al., 2014). This agent is known to promote cellular uptake, reduce fibrosis and modulate wound-healing response to implants (Ghatnekar et al., 2009; Soder et al., 2009), in turn enhancing GJIC. Under physiological conditions, the binding of the partner proteins is associated with gap junction remodeling and cellular communication during wound healing (Soder et al., 2009).

Acute application of αCT1 at the implant site produced therapeutic effects similar to those of Cx43 AsODN, including reduced neutrophil recruitment, increased tissue capsule vascularity and decreased fibrosis (Soder et al., 2009; Grek et al., 2014). Currently, clinical trials are being conducted to examine the effects of a topical formulation of αCT1 in laparoscopic surgical wounds, diabetic foot and venous leg ulcers. There are some mild reversible side effects above the maximum tolerated dose, such as piloerection, weakness, abnormal gait and breathing patterns (Grek et al., 2014). Cx43 knockout in mice led to altered expression of multiple testicular genes (Giese et al., 2012) and Cx43 in bone marrow plays an important role in hematopoietic regeneration (Presley et al., 2005). Therefore, tissue-specific targeting of connexins is needed to prevent side effects affecting other systems for successful clinical applications in the future. Non-pharmacological approaches such as the use of biomaterials have also been shown to improve wound healing. For example, bioactive glass enhances wound healing via different mechanisms. It contains ion extracts that can reduce the open probability of hemi-channels in endothelial cells during the injury phase. In the migration and proliferation stages, it can stimulate endothelial cell migration thereby upregulating growth factors, e.g., vascular growth factor, that promote angiogenesis (Li et al., 2016).

## Non-healing diabetic wounds

**Figure 4:**
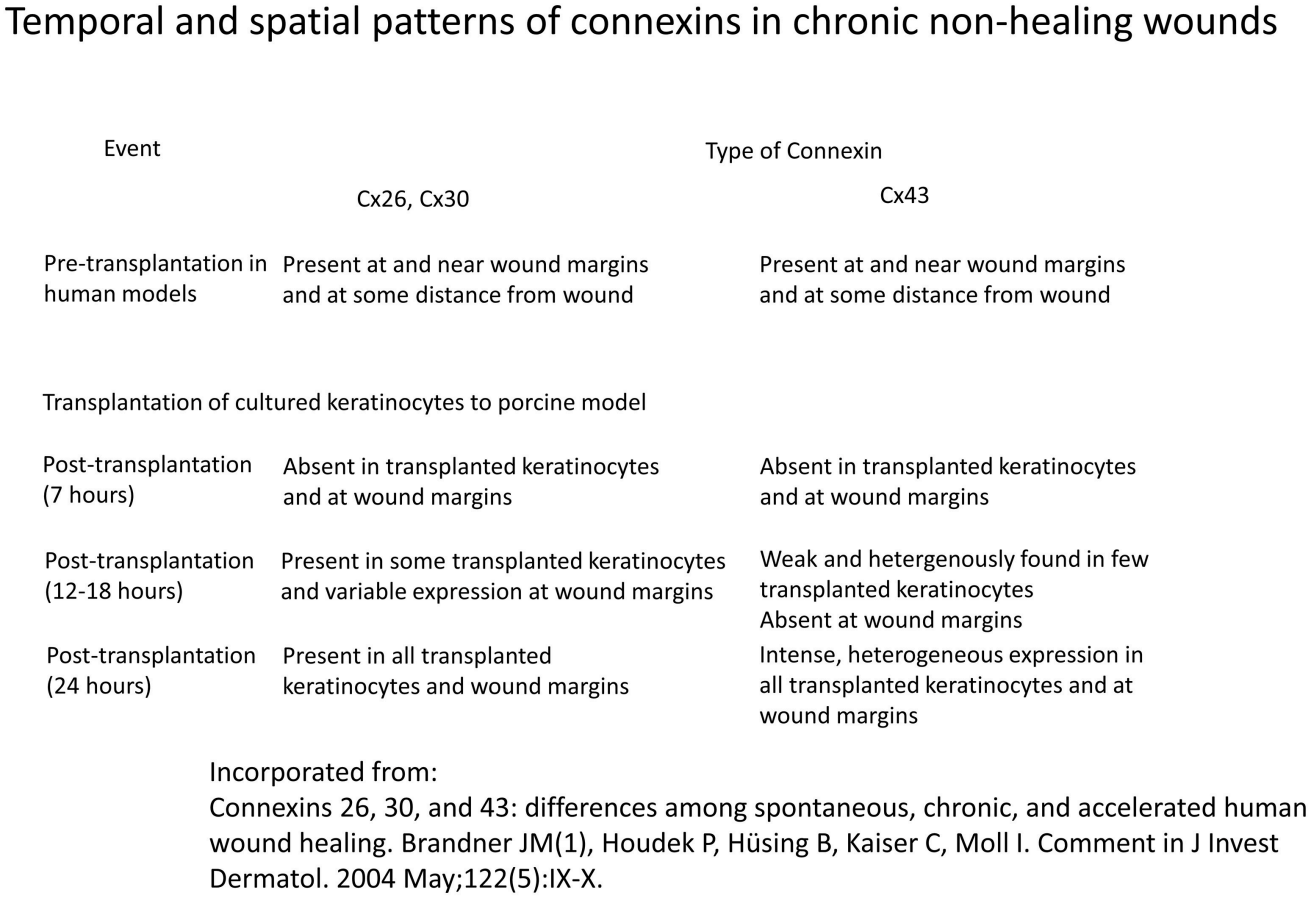
**Temporal and spatial patterns of connexins in chronic non-healing wounds Information derived from Brandner et al. (2004)**.

Diabetic wounds are known to heal with great difficulty, often resulting in ulcer formation. Furthermore, the injury size by a given insult is increased by diabetes (Palatinus and Gourdie, 2016). In non-healing wounds, Cx43 has been detected in wound margins in the vast majority of subjects, and at the periphery of wound site in all of the cases (Figure 4; Brandner et al., 2004). Some investigators have hypothesized that the cytoplasmic tail of Cx43 may bind to and interact with certain integral membrane and cytoskeletal proteins to modulate cell adhesion, cytoskeletal dynamics and ultimately, cell migration (Duffy et al., 2002; Gourdie et al., 2006; Becker et al., 2012).

Previous studies have demonstrated hyperglycaemia-induced, PKC-mediated Cx43 phosphorylation, leading to proteosomal degradation (Sato et al., 2002; Fernandes et al., 2004; Lin et al., 2006). It also depressed Cx43 gene expression and inhibited GJIC activity in cultured vascular smooth muscles. These changes were associated with alterations in connexin synthesis, phosphorylation, function and degradation. Prolonged hyperglycaemia resulted in damage to peritoneal mesothelial cells and impaired intercellular adhesion. The water soluble inducer of cellular differentiation, hexamethylene bisacetamide, reversed these pathological changes and upregulated gap junctions, thereby protecting peritoneal structural integrity (Ogawa et al., 2001). Previous studies on the relationship between Cx43 and diabetic vasculature have demonstrated a general trend of reduced Cx43 and depressed gap junction communication (Li et al., 2003; Makino et al., 2008). This was proposed to be the pathological mechanism underlying the development of macroangiopathy in diabetic patients (Kuroki et al., 1998). Many studies have demonstrated a prolonged inflammatory phase in diabetic wounds, leading to a delay in granulation tissue formation and hence significantly delaying resolution (Mustoe, 2004; Wang et al., 2007; Dinh et al., 2012). Other studies have shown that in diabetic wounds, healing is arrested in the proliferative phase with an excess of matrix proteins, potentially resulting in non-healing wounds (Falanga, 2005).

Wound healing in diabetes has been studied further in streptozotocin-induced diabetic rats (Wang et al., 2007). In the diabetic state, Cx26 and Cx43 expression and communication in the intact epidermis were all reduced, whereas Cx43 was upregulated in the intact dermis. Connexin expression in wound healing also differed between diabetes and controls. Thus, Cx43 was upregulated in a thickened bulb of keratinocytes at the wound site within 24 h, whereas it was downregulated in controls. The effects of Cx43-specific antisense gel have been tested on diabetic wounds by direct application, resulting in Cx43 upregulation and increased the rate of re-epithelialization.

The Cx43 mimetic peptide, αCT1, was shown to significantly accelerate closure of diabetic foot ulcers and increase the incidence of complete closures (Grek et al., 2015). In this study, no adverse events or cases of immunogenicity were reported, suggesting that this agent could be safely applied in humans. However, the improvement in endpoints may partly be due to better compliance, since treatment was given in a study center on a weekly basis, compared to self-administration in previous studies (Margolis et al., 2002; Balingit et al., 2012; Grek et al., 2015). A larger sample size will be needed in future studies along with increased diversity in terms of ethnicity and gender (Grek et al., 2015).

Another connexin 43 mimetic peptide, Gap27, was shown to enhance migration of keratinocytes and fibroblasts, accelerating wound healing in different mouse models (Pollok et al., 2011). Interestingly, there was a discrepancy in the responsiveness to Gap27 treatment between diabetic and non-diabetic cells (Pollok et al., 2011). Diabetic cells were immune to the migration-enhancing effects of Gap27. This may be related to a different microenvironment of the diabetic wound, such as hypoxia, the presence of pro-inflammatory mediators, high glucose levels together with an excess of matrix metalloproteinases (MMPs) relative to tissue inhibitors of MMPs (TIMPs) (Muller et al., 2008). These factors must therefore be considered when developing connexin modulator-based treatments to ensure their efficacy is not reduced (Pollok et al., 2011).

## Pressure ulcers

The National Pressure Ulcer Advisory Panel (NPUAP) defines a pressure ulcer as an area of unrelieved pressure over a defined area, usually over a bony prominence, resulting in ischemia, cell death, and tissue necrosis. More recent studies have shown that they can also be caused by ischaemia-reperfusion damage due to repeated pressure applied to the skin (Peirce et al., 2000). An *in vitro* model of ischemia-reperfusion injury in fibroblasts demonstrated increases in Cx43 levels, hemi-channel activity and cell death (Pringle et al., 1997). Gap27 exerted concentration-dependent effects: at high levels it significantly reduced Cx43 levels and GJIC, in turn reducing fibroblast cell death (Glass et al., 2015). In other studies, Gap27 was found to increase phosphorylation of S368 without altering the level of Cx43 (Evans and Boitano, 2001). It was suggested that Gap27 prevents the death of a number of cell types, including cardiomyocytes, cortical astrocytes and neurons, by blocking Cx43 opening during reperfusion (Thompson et al., 2006; Clarke et al., 2009; Orellana et al., 2010), as well as preventing “bystander effect” of cell death induction of healthy cells in close proximity (Mao et al., 2009; Danesh-Meyer et al., 2012; Zhang et al., 2013). Further investigations into the potential of Gap27 and other connexin modulators for clinical use will be the next step in improving treatment options for pressure ulcers.

## Venous leg ulcers

Patients with chronic venous insufficiency are prone to the formation of venous leg ulcers. Impaired wound healing is attributed to continuous inflammation in extracellular matrix accompanied by fibroblast and keratinocyte dysfunction (Brandner et al., 2004; Charles et al., 2008; Ongstad et al., 2013; Kim et al., 2014). Current treatment protocol includes antiseptic use, wound dressing and limb compression (O'Meara et al., 2009). A randomized trial was conducted to investigate the beneficial effects of adding αCT1 to the conventional treatment protocol (Ghatnekar et al., 2015). This approach significantly enhanced wound closure of the ulcers, resulting in a reduction of median healing time from 12 to 6 weeks compared to the standard treatment (Ghatnekar et al., 2015). However, there is limited information regarding prior treatments received, recurrence rate, and patient compliance (Ghatnekar et al., 2015). Therefore, further studies with a run-in period and an extended follow-up along with comparisons of different delivery vehicles are required to further establish the efficacy of αCT1 in wound healing of venous leg ulcers (Ghatnekar et al., 2015).

## Corneal wound healing

The corneal epithelium consists of four to six layers of non-keratinized stratified squamous epithelial cells on a uniform basement membrane (Kenyon, 1979; DelMonte and Kim, 2011). Superficial cell layers have microvilli and microplicae for metabolite transportation and tear film adhesion, whereas the basal columnar layers are more metabolically active (Lu et al., 2001). At least eight Cx isoforms (Cx26, Cx30.3, Cx31, Cx31.1, Cx32, Cx43, Cx45, and Cx50) have been identified in the human corneal epithelium (Yuan et al., 2009; Zhai et al., 2014).

Corneal wound healing shares some similarities with skin healing (Moore et al., 2013). Epithelial healing starts with a non-mitotic wound coverage phase by cellular migration and spreading over the defect, followed by mitosis of epithelial cells (DelMonte and Kim, 2011). Stromal injuries induce migration and activation of keratocytes and subsequently stromal remodeling and fibrosis (Fini and Stramer, 2005). Endothelial trauma is resolved firstly by migration and coverage of adjacent endothelial cells, then return of normal tight junction function and lastly endothelial cell remodeling (Watsky et al., 1989; DelMonte and Kim, 2011). Pre-clinical experiments suggest that connexins play a role in corneal wound healing. In rabbit cornea after excimer laser photorefractive keratectomy, Cx43 and Cx26 were found to be upregulated (Ratkay-Traub et al., 2001). This is in corroboration with human findings where increased expressions of Cx26, Cx31.1, and Cx43 were detected in chemically burned and infected corneas (Zhai et al., 2014).

The commonest clinical method for cornea regeneration involves application of the amniotic membrane, although it has disadvantages such as donor dependent differences (Tsai et al., 2015). Other efforts have focused on the use of Cx43 mimetic peptides to promote corneal wound healing. Epithelial-to-mesenchymal transition (EMT) is a cellular process involving mobilization of sedentary cells to areas needing repair (Leopold et al., 2012) and is thought to play a role in the cornea during healing (Lee et al., 2012; Kowtharapu et al., 2014). An extended-release preparation of αCT1 using microcapsules was compared with a single high initial dose of αCT1 or pluronic gel vehicle (Moore et al., 2013). Wound closure analysis showed that healing time was significantly reduced in rat cornea treated with αCT1 microcapsule compared with other two regimes, showing a 14.55% improvement compared to pluronic gel treatment, while a single high αCT1 dose resulted in only a 12.56% improvement. However, why αCT1 treatment produced higher closure rate at 24–72 h even though the microencapsulated αCT1 gives a higher percentage healing overall is unclear (Moore et al., 2013). Three different genes were analyzed with RT-PCR to study their short and long-term effects on corneal healing. Cx43 expression demonstrated a biphasic response, and was downregulated in αCT1 treatments in day 21, contrary to that in control group. ZO-1 was downregulated throughout the length of study for all set-ups. Keratin 19 (Krt19), a corneal epithelial progenitor and stemness marker gene that is thought to be downregulated during EMT, was significantly elevated after 24 h then downregulated in αCT1-treated cornea (Moore et al., 2013).

A later study by the same group examined the effects of the same αCT1 preparations in type I diabetic corneal wound healing using a streptozotocin type 1 diabetic rat model (Moore et al., 2014). It was found that αCT1, whether applied directly as pluronic gel solution or delivered in a sustained manner using microcapsules, accelerated wound closure significantly at days 1 and 3, with the latter producing the most rapid effects. The probability of wound healing rate calculated using a modified Kaplan-Meier method indicated that αCT1, especially the microcapsule form, consistently improved corneal wound healing (Moore et al., 2014). The mechanism involves reduced inflammation as reflected by downregulation of the markers, Interferon Inducible T-Cell Alpha Chemoattractant and Tumor Necrosis Factor alpha markers (Moore et al., 2014). TGF-β was the only gene upregulated throughout all time points. All three isoforms of TGF-β are expressed in the cornea, and play a role in corneal development and wound healing (Jester et al., 1997; Saika, 2004). The effects of TGF-β in suppressing inflammation, promoting fibroblast proliferative activity and ECM deposition are well-established (Carrington et al., 2006). These findings provide further evidence for the role of TGF-β in corneal re-epithelialisation, through induction of keratocyte transformation into fibroblasts during wound healing (Stramer et al., 2003). Krt19 level was again measured and initially downregulated followed by upregulation at later time points (Moore et al., 2014).

Moreover, Krt19 was negatively correlated to EMT (Aomatsu et al., 2011). It is not known whether the upregulations at initial and later stages were due to (1) artifacts; (2) an unknown underlying mechanism that may have caused an initial transient spike; or (3) hyperglycemia which may be responsible for inducing elevation of Krt19 level. Another study found that Snail gene overexpression during corneal wound healing induced upregulation of gap junction proteins (e.g., Cx43) and downregulation of stemness markers (e.g., Krt19) in mice (Aomatsu et al., 2012). The roles of Krt19 and Cx43 in differentiation and migration may provide further insight into the interplay between connexins and stemness markers in EMT. Based on the current evidence, it is suggested that the changes in phenotypic expression of stemness and differentiation markers in wounded corneal epithelium may be responsible for healing through EMT (Aomatsu et al., 2012). This could be achieved by arresting proliferation (Liu et al., 2010), remodeling of the cytoskeleton and enhancing migration (Thiery, 2002; Chen et al., 2004; Aomatsu et al., 2012). These four physiological processes may represent targets for modulating connexins to achieve therapeutic healing effects in the future.

## Summary

Connexins are ubiquitously expressed with tissue-specific subtypes. Their expression patterns in different diseases are now better characterized. Their ability to regulate immune responses, cell proliferation, migration and apoptosis makes them attractive therapeutic targets to promote the skin wound healing, diabetic and venous ulcers, as well as cornea healing. Novel approaches involve the use of antisense technology to reduce connexin expression, or synthetic mimetic peptides to reduce the function of connexins, which have demonstrated successes in pre-clinical disease models, with great potential in the future for clinical applications.

## Author contributions

PW: Design of manuscript; drafted and critically revised the manuscript for important intellectual content; preparation of figures. TT: Drafted and critically revised the manuscript for important intellectual content. CC: Drafted and critically revised the manuscript for important intellectual content. VL: Drafted and critically revised the manuscript for important intellectual content. YC: Drafted and critically revised the manuscript for important intellectual content. TL: Critically revised the manuscript for important intellectual content. WW: Critically revised the manuscript for important intellectual content. GT: Design of manuscript; drafted and critically revised the manuscript for important intellectual content.

### Conflict of interest statement

The authors declare that the research was conducted in the absence of any commercial or financial relationships that could be construed as a potential conflict of interest. The reviewer MT and handling Editor declared their shared affiliation, and the handling Editor states that the process nevertheless met the standards of a fair and objective review.
